# Two-dimensional gold nanostructures with high activity for selective oxidation of carbon–hydrogen bonds

**DOI:** 10.1038/ncomms7957

**Published:** 2015-04-22

**Authors:** Liang Wang, Yihan Zhu, Jian-Qiang Wang, Fudong Liu, Jianfeng Huang, Xiangju Meng, Jean-Marie Basset, Yu Han, Feng-Shou Xiao

**Affiliations:** 1 Key Lab of Applied Chemistry of Zhejiang Province, Department of Chemistry, Zhejiang University, Hangzhou 310028, China; 2 Advanced Membranes and Porous Materials Center, Physical Sciences and Engineering Division, King Abdullah University of Science and Technology, Thuwal 23955-6900, Kingdom of Saudi Arabia; 3 Key Laboratory of Interfacial Physics and Technology, Shanghai Institute of Applied Physics, Chinese Academy of Sciences, Shanghai 201800, China; 4 Research Center for Eco-Enviromental Sciences, Chinese Academy of Sciences, Beijing 100085, China; 5 KAUST Catalysis Center, King Abdullah University of Science and Technology, Thuwal 23955-6900, Kingdom of Saudi Arabia

## Abstract

Efficient synthesis of stable two-dimensional (2D) noble metal catalysts is a challenging topic. Here we report the facile synthesis of 2D gold nanosheets via a wet chemistry method, by using layered double hydroxide as the template. Detailed characterization with electron microscopy and X-ray photoelectron spectroscopy demonstrates that the nanosheets are negatively charged and [001] oriented with thicknesses varying from single to a few atomic layers. X-ray absorption spectroscopy reveals unusually low gold–gold coordination numbers. These gold nanosheets exhibit high catalytic activity and stability in the solvent-free selective oxidation of carbon–hydrogen bonds with molecular oxygen.

## Abstract (web summary)

There is interest in the study of two-dimensional materials for catalysis. Here, the authors report the fabrication of two-dimensional gold nanostructures, using a layered double hydroxide as a stabilizing template, and evaluate the catalytic performance of the composite for solvent free selective oxidation.

## Graphical abstract

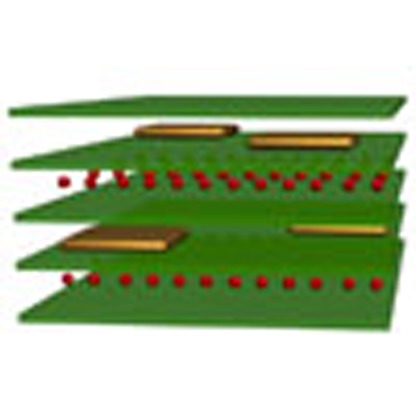

Two-dimensional (2D) materials with single to a few atomic layer thicknesses, as represented by graphene, have attracted enormous research interest in the last decade, owing to their fascinating electronic, magnetic, optical and catalytic properties^1^,^2^,^3^,^4^,^5^,^6^,^7^,^8^,^9^,^10^,^11^,^12^,^13^,^14^,^15^. In comparison with lamellar-structured graphite or metal dichalcogenides (for example, MoS_2_), which can readily be prepared in the form of 2D materials by physical^16^,^17^,^18^ or chemical exfoliation^19^,^20^,^21^,^22^, noble metals are more difficult to be fabricated into 2D nanostructures, especially by wet chemical synthesis, because of their highly isotropic lattice symmetries. Some anisotropic metallic colloidal nanosheets (NSs) have been synthesized in solution using various capping agents via direct^23^,^24^,^25^,^26^,^27^,^28^,^29^,^30^,^31^ or secondary growth^31^, but with few exceptions^27^, they have thicknesses of several nanometers or even greater and cannot be classified as 2D materials in a strict sense. It is known that with noble metals (for example, Au) extraordinary catalytic activity occurs as a consequence of the quantum size effect when the catalyst contains only two to three atomic layers^32^,^33^,^34^. 2D Au nanostructures have been prepared on well-defined crystal surfaces by physical deposition methods^32^,^35^,^36^. They have controllable thicknesses within the range of a few layers of atoms but also limited size (several nanometers) in the other two dimensions, and they are therefore usually referred to as ‘islands'^32^,^35^,^36^. It was reported that 2D Au islands deposited on the surface of graphene/Ru(0001) could promote CO adsorption and potentially catalyze the CO oxidation at very low temperature^35^; likewise, 2D Au islands prepared on the TiO_2_(110) surface showed greatly enhanced molecular binding especially at the edge sites^32^. In general, 2D metallic materials are supposed to show distinct molecular activation ability and catalytic behaviours, considering that they have unusual electronic structures as well as a large proportion of low-coordinated atoms. However, only molecular adsorption properties have thus far been investigated for 2D Au catalysts^32^, due to the difficulty in preparing them on the large scale^35^,^36^ and their weak stability^29^. Efficient synthesis of stable 2D Au catalysts remains unattained.

Layered double hydroxides (LDHs) belong to a class of lamellar-structured clay with a general formula of M(II)_1-x_M(III)_x_(OH^−^)_2_(A^n−^)_x/n_·zH_2_O, where the positive charges of the cationic layers made of edge-shared metal (M(II) and M(III)) hydroxide octahedra are balanced by the interlayered anions (A^n−^)^37^. One attractive feature of LDH materials is that the interlayered anions have considerable freedom of movement to be substituted by other types of anions via ion exchange^38^,^39^,^40^,^41^,^42^.

In this work, we exploit the anion-exchange capability of LDH to introduce Au precursors (AuCl_4_^−^) into the interlayer space of Mg/Al-LDH for subsequent chemical reduction to prepare a Au/LDH hybrid material (Fig. 1). In this way, we successfully synthesize ultra-thin 2D Au NSs between the metal hydroxide layers of LDH, which not only provide a confined-space effect to achieve controllable crystal growth, but also stabilize the resulting Au NSs. A detailed study by high-resolution electron microscopy illustrates that the as-prepared Au NSs are single crystalline with (001) basal planes and {100}-type edges, and that they are ultra-thin down to a few atomic layers. These 2D Au nanostructures exhibit excellent catalytic activity and stability towards the selective and solvent-free oxidation of C–H bonds using molecular oxygen as the oxidant.

## Results

### Synthesis and characterization

**Figure 2:**
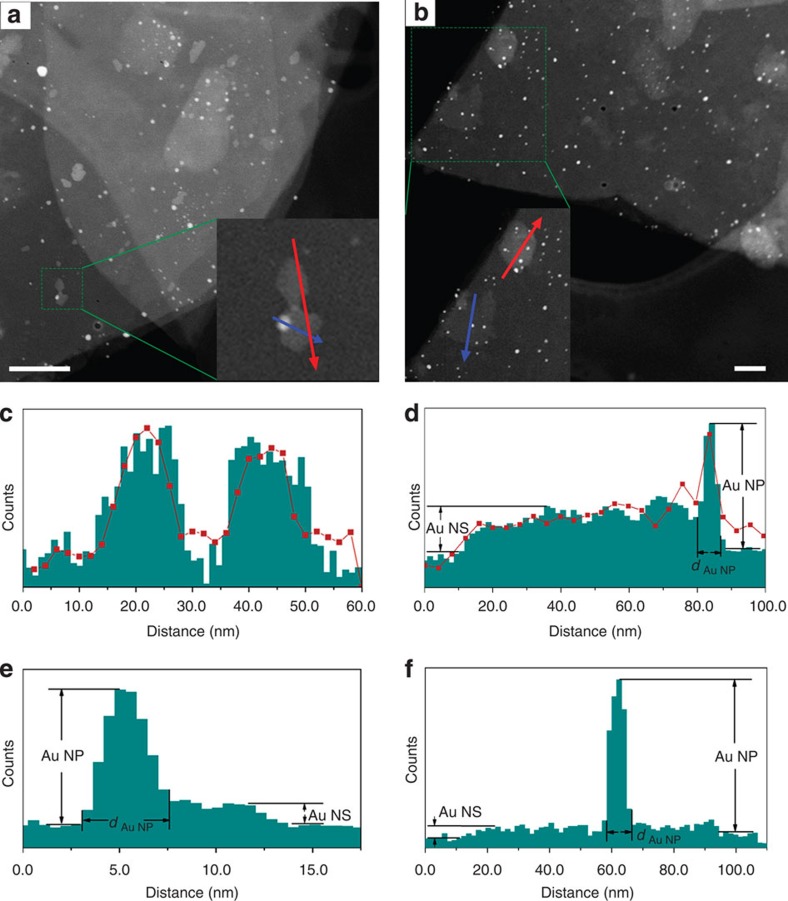
Two sets of STEM/EDX analysis of the Au/LDH hybrid. (**a**,**b**) STEM images in which both Au NPs and Au NSs are observed in the LDH substrate. Scale bar, 100 nm for **a** and 50 nm for **b**. The selected regions are enlarged as the insets, and line scanning was performed over the NSs following the two arrows. (**c**,**d**) The normalized STEM intensity (green histogram) and the Au M-edge EDX intensity (red curves) line profiles, collected along the red arrows in **a**,**b**, respectively. (**e**,**f**) The STEM intensity line profiles collected along the blue arrows in (**a**,**b**), respectively. The EDX results confirm that the NSs are made of Au; the thickness of the Au NSs can be determined based on the STEM intensity by using Au NPs as a reference.

The as-synthesized Au/LDH hybrid contained 0.13 wt% of Au, as determined by inductively coupled plasma optical emission spectrometry (ICP-OES). Such a small loading of Au did not give rise to discernible change in the powder X-ray diffraction pattern (XRD) of LDH (Supplementary Fig. 1). However, the Au species could be clearly distinguished from the LDH substrate in the high-angle annular dark-field (HAADF) scanning transmission electron microscopy (STEM) images by the Z-contrast, which exhibited two kinds of typical morphologies: nanoparticles (NPs) in a size range of 2–10 nm; and irregular NSs with sizes ranging from several nanometers to tens of nanometers (Fig. 2, Supplementary Fig. 2). The NSs show apparently weaker contrast than even the smallest NPs, implying their ultra-thin nature. Assuming that Au NPs are spherical (that is, their ‘thickness' is equal to their ‘diameter'), the thickness of a Au NS can be approximately determined based on its contrast relative to that of a AuNP, because in HAADF-STEM the image intensity is roughly proportional to the specimen thickness for a given material composition^43^,^44^. One example is shown in Fig. 2a, in which the thickness of a Au NS was determined to be ∼1.0 nm from the image intensity by using an adjacent AuNP (4.5 nm) as the reference, and energy dispersive X-ray spectroscopy (EDX) line scanning confirmed that the NSs are made of Au (Fig. 2a,c,e, Supplementary Fig. 3). Figure 2b shows another two NSs with larger lateral dimensions. With the method described above, the NS with brighter contrast was determined to be 1.6-nm thick, and its composition was confirmed by EDX to be Au (Fig. 2d). Although the EDX signal from the NS with lower contrast was too weak to be detected, it is presumed to be Au as well, considering its similarity in morphology to the thicker sheets. Accordingly, its calculated thickness based on the HAADF-STEM image intensity is 0.2–0.4 nm, which corresponds to only 1–2 atomic layers (Fig. 2f). We have randomly examined over 20 NSs and found that the thickness of a large portion of them is <1 nm. These NSs feature sub-nanometer thicknesses and large basal surfaces, which have not been simultaneously achieved in previous 2D Au nanostructures.

**Figure 3:**
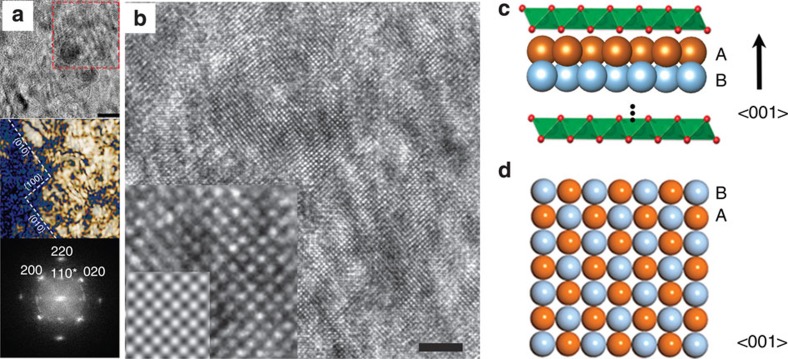
HRTEM analysis of 2D Au NS. (**a**) HRTEM image taken at the edge of a 2D Au NS with scale bar at 5 nm, and Bragg-filtered image derived by inverse FFT of (200) and (020) reflections in the FFT diffractogram. The boundaries of the Bragg-filtered image were further enhanced by a Sobel filter. (**b**) Enlarged image of the highlighted region in **a** with scale bar at 2 nm. The inset is a further enlarged image to show atomic columns in comparison with a simulated HRTEM image (bottom left corner); the simulation was based on a three-layer [001]-oriented Au structural model (300 kV; Cs: 1.2 mm; Cc=1.2 mm; Δ*E*=0.7 eV; Δ*f*=−65 nm). Schematic illustrations of (**c**) the AB-stacked [001]-oriented 2D Au NS intercalated in the LDH, and (**d**) 2D Au NSs projected along the [001] axis, where gold atoms in different atomic layers (**a**,**b**) are represented by spheres in different colours.

Figure 3 shows a high resolution transmission electron microscopy (HRTEM) image of a Au/LDH hybrid, in which lattice fringes are clearly observed over a large area (>100 nm^2^). We demonstrated in a control experiment that the crystalline structure of the LDH was immediately amorphized, as evidenced by the rapid disappearance of reflections in the electron diffraction pattern, under similar TEM imaging conditions. Therefore, the observed lattice fringes must be associated with the 2D Au NS. The corresponding fast Fourier transform (FFT) indicates that the Au NS is single crystalline and [001] oriented. Notably, the (110) reflections that are forbidden for bulk Au appear in the FFT (marked with an asterisk, Fig. 3a), which is a characteristic phenomenon for 2D materials due to the loss of symmetry elements in 1D (ref. 27). We simulated HRTEM images for the [001]-oriented Au NSs of different thicknesses starting from a single monolayer. The results show that the (110) reflections appear in the FFTs when the NS contains an odd number of atomic layers and their intensity rapidly decreases as the layer number increases (Supplementary Fig. 4). By comparing the experimental and simulation results, we determined that the observed Au NS has an average thickness of three atomic layers based on the intensity ratio of reflections (*I*_(110)_/*I*_(200)_). As visualized by the Bragg-filtered HRTEM image, the lateral boundaries of the 2D Au NS mainly comprise {100}-type zigzag edges (Fig. 3a).

It is naturally expected that a lateral image of 2D Au NSs allows a better understanding of their structures and direct measurement of their thicknesses. Therefore, we used focused-ion-beam technique to cut a thin (∼ 50 nm) slice out of a Au/LDH hybrid particle with the cutting direction perpendicular to the basal plane of LDH, to be able to observe the cross-sections of 2D Au NSs with HAADF-STEM. The acquired images indeed show good evidences for the presence of 2D Au NSs intercalated within the LDH layers (Supplementary Fig. 5). However, the ultra-thin nature and the consequent instability (on the irradiation of ion/electron beams) of the Au NSs inhibited more in-depth analysis, as the structure evolved during the focused-ion-beam and STEM imaging processes even with extremely low doses. The HRSTEM images of this direction along with detailed discussions can be found in the Supplementary Information (Supplementary Fig. 5).

(S)TEM characterization explicitly demonstrates the intercalation of sub-nanometer-thick Au NSs in the LDH substrate. It is worth noting that these Au NSs are unique not only in thickness but also in orientation. Unlike previously reported Au NSs that are usually [111] oriented^28^,^30^, the Au NSs in the Au/LDH hybrid are [001] oriented, which may arise from the preferential interaction of the cationic LDH framework and the Au (001) surfaces. We attempted to isolate the Au NSs by treating the hybrid with HCl, but we found that the removal of LDH led to the transformation of Au NSs into NPs (only bulky Au NPs were observed in the final product). This result indicates the crucial stabilization effect of LDH on these anomalously orientated ultra-thin Au NSs. The metal–substrate interaction in the Au/LDH hybrid was characterized using X-ray photoelectron spectroscopy (XPS) by comparison with two reference materials (Supplementary Fig. 6), silica-supported Au NPs and LDH-supported Au NPs (Au NPs are deposited on the external surface of LDH). Silica-supported Au NPs show a binding energy of Au4f_7/2_ at 84.1 eV, which is characteristic of metallic Au species^45^. LDH-supported Au NPs have a smaller Au4f_7/2_ binding energy of 83.8 eV. Remarkably, the Au4f_7/2_ binding energy measured for the Au/LDH hybrid is as low as 83.1 eV, corresponding to a large red shift of 1.0 eV relative to metallic Au. This reveals that the intercalated 2D Au NSs are highly negatively charged and chemically bonded with the cationic layers of LDH.

**Figure 4:**
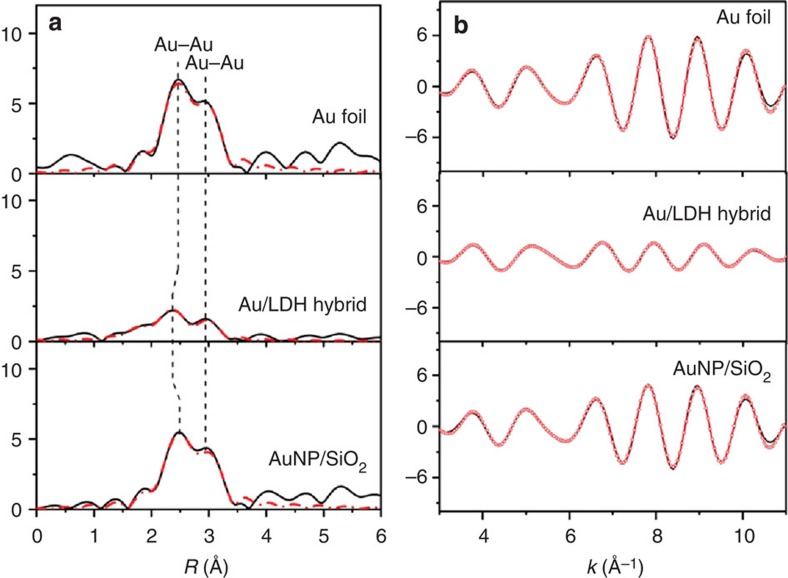
EXAFS spectra of the Au-L_III_ edge in various Au samples. (**a**) Fourier transforms of filtered *k*^3^·*χ*(*k*) into the R space, where the red dashed lines correspond to the curve-fitting results; (**b**) filtered *k*^3^·*χ*(*k*) in the *k* range of 3–11 Å^−1^, where the red dotted lines correspond to the curve-fitting results.

The average coordination environment of Au in the Au/LDH hybrid was analysed based on the extended X-ray absorption fine structure (EXAFS) of the Au-L_III_ edge (Fig. 4). In comparison with Au foil (bulk Au) or SiO_2_-supported Au NPs, the Au/LDH hybrid has much less intense peaks associated with the Au–Au first-shell coordination. The derived Au–Au coordination number and bond distance are 6.9±0.6 Å and 2.80±0.01 Å, respectively, for the Au/LDH hybrid (Supplementary Table 1); and these values are markedly smaller than those of Au foil (11.2±0.9 and 2.85±0.01) and silica-supported Au NPs (10.1±0.8 and 2.86±0.01). These results provide further evidence for the presence of 2D Au nanostructures with atomic thickness in the Au/LDH hybrid that is responsible for the unusually low coordination number of Au. In addition, the Au–O coordination with a relatively small coordination number (0.7±0.3) was also observed in the hybrid, suggesting a strong interaction between the surface atoms of Au NSs and the LDH cationic layers.

### Catalyst evaluation

A series of supported Au catalysts was prepared with comparable Au loading amounts (0.1–0.2 wt%). The statistical analysis based on TEM images shows that, despite slight variations from sample to sample, all the catalysts have a similarly broad size distribution of Au NPs (2–10 nm) with the majority in the range of 3–6 nm (Supplementary Figs 7 and 8). There is no remarkable difference in particles sizes among different catalysts including the Au/LDH hybrid (only taking Au NPs into account). The catalysts were tested for the selective oxidation of ethylbenzene and toluene by molecular oxygen under solvent-free condition, where the ethylbenzene and toluene act as model molecules with secondary and primary C–H bonds, respectively. As summarized in Table 1, the catalysts containing Au NPs on conventional supports exhibit fairly low activity for these reactions: AuNP/SiO_2_ and AuNP/FeO_x_ converted <7% of ethylbenzene, while the AuNP/C catalyst was nearly inactive. In contrast, the Au/LDH hybrid exhibited much higher activity, giving an ethylbenzene conversion of 39.2%. Even when air was used as the oxidant, the conversion was as high as 32.5%. The selectivity of acetophenone was >90% in this system (Table 1). When toluene was used as the reactant, most of the tested catalysts were inactive, which is in good agreement with the results in literature that the sole Au catalysts failed to catalyse the selective oxidation of toluene^46^,^47^. Very interestingly, the Au/LDH hybrid exhibited a noticeable conversion of 9.2%. Control experiments were carried out using LDH (without loading Au) and Au NP-deposited LDH (AuNP/LDH) as catalysts, during which the former was essentially inactive for both ethylbenzene and toluene oxidation and the latter was performed similarly to the Au NPs on conventional supports (Table 1). These results demonstrated that the high catalytic activity of the Au/LDH hybrid comes mainly from the 2D Au NSs. Considering the configuration of the Au NSs (sandwiched within the LDH layers), the edges of the 2D Au NSs would act as major catalytic active sites for the reactions.

**Figure 5:**
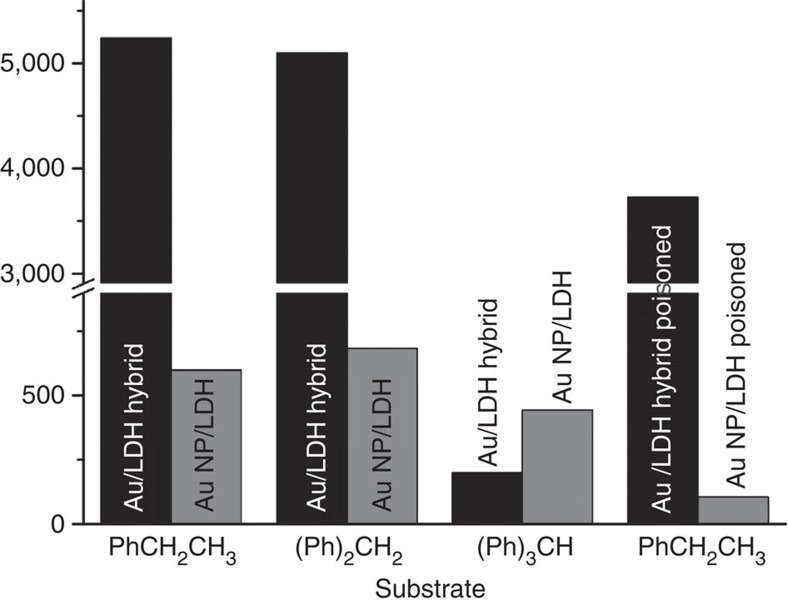
Turnover frequencies of Au catalysts for the catalytic oxidation of various substrates. The reaction conditions are the same as specified in Table 1. The TOF values were normalized by the total amount of Au in the catalyst during the reaction time of 15 min.

We used molecular probes with different sizes, including ethylbenzene, diphenylmethane and triphenylmethane, to investigate the catalytic roles of the 2D Au NSs in the Au/LDH hybrid. Figure 5 shows the turnover frequency (TOF) of various substrates over the Au/LDH hybrid and AuNP/LDH catalysts, which were calculated based on the overall number of Au atoms in each catalyst. Clearly, the Au/LDH hybrid is much more active than the AuNP/LDH in the catalytic oxidation of both ethylbenzene (TOF: 5240, h^−1^ versus 600 h^−1^) and diphenylmethane (TOF: 5100, h^−1^ versus 685 h^−1^). When the bulky triphenylmethane was used as the substrate, however, the Au/LDH hybrid exhibited dramatically lower activity (TOF: 200 h^−1^), whereas AuNP/LDH essentially retained its activity for small substrates (TOF: 443 h^−1^). In the AuNP/LDH catalyst, the Au NPs (catalytic active sites) reside on the external surface of the support with little diffusion limitation, and therefore it exhibits similar reaction rates for different substrates; in the case of the Au/LDH hybrid, however, bulky substrates have difficulty in diffusing into the interlayer regions of LDH and thus have limited contact with the edges of Au NSs, resulting in slower reactions. These results also suggest that the intercalated 2D Au NSs make a greater contribution to the catalytic activity of the Au/LDH hybrid than do the Au NPs on the LDH surface.

To directly probe the activity of 2D Au NSs in the Au/LDH hybrid catalyst, we selectively poisoned the Au NPs on the surfaces of LDH by capping them with polyvinylpyrrolidone (PVP, molecular weight at ∼58,000) to inhibit access of the reactant molecules^48^. It is conceivable that the Au NSs would not be poisoned because PVP molecules are too large to enter the interlayer spaces of LDH. This poisoning strategy proved to be effective, as it successfully deactivated the AuNP/LDH catalyst. As shown in Fig. 5, on the poisoning treatment, the TOF value of AuNP/LDH for the oxidation of ethylbenzene decreased by ∼80% (from 600 to 105 h^−1^). In contrast, the Au/LDH hybrid catalyst retained high activity (TOF at 3724, h^−1^) after the same treatment. This result unambiguously proves that the superior catalytic activity of the Au/LDH hybrid is largely because of the intercalated ultra-thin Au NSs. Since the exact weight percentage of Au NSs in the Au/LDH hybrid cannot be determined due to the presence of Au NPs, the TOF discussed above was calculated based on the total number of Au atoms. The real TOF of the 2D Au NSs should have an even higher value. Our preliminary calculation results based on a simplified system suggest that the negatively charged edge sites of 2D Au NSs synergize with the neighbouring hydroxyl groups of the LDH cationic layer to facilitate the adsorption and activation of oxygen molecules (Supplementary Fig. 9, Supplementary Table 3). However, it should be pointed out that the adsorption of oxygen molecules only represents one of the several key steps of the reaction. More intensive studies are needed to better understand the origin of the high oxidation activity of the Au/LDH hybrid catalyst.

The Au/LDH hybrid catalyst is reusable. After each reaction run, it can be easily recycled by filtration with negligible Au leaching as confirmed by ICP-OES. Consequently, it exhibits constant catalytic performance during continuous reaction cycles. When used for the aerobic oxidation of ethylbenzene, for example, it gave stable conversion of ethylbenzene (∼38%) and selectivity of acetophenone (∼90%) in five reaction runs (Supplementary Fig. 10a). Furthermore, it is worth noting that the reaction rate of ethylbenzene conversion at the beginning (15 min) of each cycle is similar (Supplementary Fig. 10b), indicating the good recyclability of Au/LDH hybrid catalyst. Moreover, the Au/LDH hybrid catalyst is thermally stable. It showed unchanged catalytic activity for ethylbenzene oxidation after being heated at 350 °C for 2 h (Table 1). We used EXAFS to identify the structural evolution of the Au/LDH hybrid on heating treatment at different temperatures, and we found that the 350 °C-treated sample retained a low average Au–Au first-shell coordination number (7.5±1.3). The 550 °C-treated sample, however, showed markedly increased Au–Au coordination numbers (10.2±1.6), suggesting the transformation of 2D Au NSs to NPs (Supplementary Fig. 11). We characterized a Au/LDH hybrid sample, which was heated at 350 °C for 2 h and then used to catalyse the ethylbenzene oxidation for 16 h, with electron microscopy. The HRTEM image reveals the presence of ultra-thin, single-crystalline and [001]-oriented Au nanostructures (Supplementary Fig. 12), providing more straightforward evidence for the retention of 2D Au NSs during thermal treatment and catalytic use. These results demonstrate that the 2D Au NSs in the hybrid has excellent thermal stability up to at least 350 °C. Further investigation indicated that the Au/LDH hybrid catalyst is generally effective for the selective oxidation of various phenylic alkanes with primary and secondary C–H bonds. In the tested reactions, which were all performed under solvent-free conditions using molecular oxygen, it consistently gave the desired products in good activities and selectivities (Supplementary Table 4). The good stability combined with the general applicability makes the Au/LDH hybrid catalyst potentially useful for wide applications in the oxidation of C–H bonds.

## Discussion

In summary, we successfully cast 2D Au NSs with thicknesses of single to a few atomic layers through confined-space synthesis using Al-Mg LDH as the host material. These ultra-thin Au NSs are negatively charged and stacked on the non-densely packed {001} family of crystal planes. In comparison with Au NPs supported on various materials, the Au NSs exhibited exceptionally high catalytic activities in the solvent-free oxidation of C–H bonds, which we attribute to the exposure of the low-coordinated edge sites. The LDH host material also stabilizes the Au NSs, making them reusable for multiple reaction runs with nearly unchanged catalytic performance. This synthetic method can be potentially used to prepare other types of 2D metallic nanocatalyst.

## Methods

### Catalyst preparation

*Synthesis of LDH*. About 30.76 g of Mg(NO_3_)_2_·6H_2_O and 15 g Al(NO_3_)_3_·9H_2_O were dissolved in 400 ml of water, followed by addition of 72 g of urea under stirring. After boiling for 8 h, precipitating at room temperature for 12 h, filtrating and washing with a large amount of water, samples of Mg-Al-LDH (molar ratio of Mg/Al at 3) were obtained.

*Synthesis of Au/LDH hybrid*. About 3 g of LDH powder was added into a 50 ml aqueous solution of HAuCl_4_ (4.8 × 10^−4^ M) and stirred for 8 h at room temperature. After filtrating, washing with a large amount of water and drying under vacuum at 60 °C overnight, 1 g of the obtained solid sample was reduced by 35 mg of NaBH_4_ to obtain metallic Au in 25 ml of anhydrous toluene and 7 ml of EtOH at room temperature for 6 h. Samples designated as ‘Au/LDH hybrid' with Au loading at 0.13 wt% (by ICP) were obtained.

AuNP/SiO_2_, AuNP/C and AuNP/FeO_x_ were synthesized by the homogeneous deposition–precipitation method. In a typical run for the synthesis of AuNP/SiO_2_, 3 g of amorphous SiO_2_ (mesoporous MCM-41) was added to a 50 ml solution of HAuCl_4_ (6.9 × 10^−4^ M) and urea (molar ratio of urea/Au at 100) and stirred at 90 °C for 4 h in a closed reactor kept away from light. Then the solid sample was filtrated, washed with large amount of water, dried at 100 °C for 12 h and calcined at 400 °C for 3 h. After treating the solid sample in anhydrous solvent of toluene and EtOH with NaBH_4_ at room temperature for 6 h, the sample designated as AuNP/SiO_2_ was obtained. The AuNP/C and AuNP/FeO_x_ samples were synthesized in the same way using activated carbon and iron oxide as supports, respectively. The Au loadings on AuNP/SiO_2_, AuNP/C and AuNP/FeO_x_ were 0.15 wt%, 0.20 wt% and 0.15 wt%, respectively.

*Synthesis of AuNP/LDH*. About 700 mg of PVP (molecular weight=58000) was added into a 50-ml aqueous solution of HAuCl_4_ (4.8*10^−4 ^M). The mixture was further stirred for 30 min under a bath of 0 °C. Then, the aqueous solution of NaBH_4_ (0.05 M, 15 ml) was added into the mixture under vigorous stirring. After stirring at 0 °C for another 2 h, 3 g of LDH powder was added into the mixture and stirred at room temperature for 3 h. Then, the mixture was stirred at 80 °C overnight to evaporate the water. Finally, the solid powder was calcinated at 500 °C for 3 h in pure oxygen and treated in anhydrous solvent of toluene and EtOH with NaBH_4_ at room temperature for 6 h. AuNP/LDH sample with the Au loading amount of 0.16 wt% was finally obtained.

### Characterization

Powder XRDs were obtained with a Rigaku D/MAX 2550 diffractometer with CuKα radiation (*λ*=0.154056, nm). The content of Au was determined from ICP with a Perkin-Elmer plasma 40 emission spectrometer. XPS spectra were performed by a Thermo ESCALAB 250, and the binding energy was calibrated by C1s peak (284.5 eV). The EXAFS of the Au-L_III_ edge in Au foil and other Au samples were measured in a fluorescence mode at room temperature on BL14W1 beam line in the Shanghai Synchrotron Radiation Facility (SSRF). The storage ring was operated at 3.5 GeV with 200 mA as an average storage current. The synchrotron radiation was monochromatized with a Si (111) double crystal monochromator. Data were analysed using Athena and Artemis from the IFeffit1.2.11 software package. XANES were normalized with edge height and then the first-order derivatives were taken to compare the variation of absorption edge energies. EXAFS oscillation, *χ*(*k*), was extracted using spline smoothing with a Cook-Sayers criterion, and the filtered *k*^3^-weighted *χ*(*k*) was Fourier transformed into R space in the *k* range of 3–11 Å^−1^ with a Hanning function window. In the curve-fitting step, the possible backscattering amplitude and phase shift were calculated using FEFF8.4 code. HRTEM imaging was carried out on a FEI-Titan ST electron microscope operated at 300 kV with a point resolution of 0.19 nm. TEM image simulation was carried out using the QSTEM code with multi-slice method.

### Catalytic tests

The solvent-free aerobic oxidation of ethylbenzene and toluene were performed in a high-pressure autoclave with a magnetic stirrer (1200, r.p.m.). Typically, the substrate, catalyst and initiator were mixed in the reactor by stirring for 1 h at room temperature. Then, the reaction system was heated to a given temperature (the temperature was measured with a thermometer in an oil bath) and oxygen was introduced and kept at the desired pressure. After the reaction, the product was taken out from the reaction system and analysed by gas chromatography (GC-14C, Shimadzu, using a flame ionization detector) with a flexible quartz capillary column coated with FFAP. The TOFs were calculated from the converted substrate per hour over per mol of Au species. The typical reaction conditions were as follows: 47 mmol of substrate, 100 mg of catalyst, 16 h of reaction time, 140 °C, oxygen pressure at 3 MPa, *t-*butyl hydroperoxide (TBHP in dodecane solution, 3 mol% based on substrate was added as the initiator) and reaction time of 15 min. The recyclability of the catalyst was tested by separating it from the reaction system by successive centrifugation, washing with a large quantity of methanol/water and drying at 80 °C for 3 h.

## Author contributions

L.W. prepared the catalyst, performed the characterizations and catalytic tests. Y.Z. performed the TEM characterization and analysed the catalyst structure. J.-Q. W. performed the EXAFS characterization and analysed the data. F.L., J.H., X.M. and J.-M.B. participated the discussion and analysis of the experimental data. L.W., Y.Z., Y.H. and F.-S.X. designed this study, analysed the data and wrote the paper.

## Additional information

**How to cite this article:** Wang, L. *et al*. Two-dimensional gold nanostructures with high activity for selective oxidation of carbon–hydrogen bonds. *Nat. Commun.* 6:6957 doi: 10.1038/ncomms7957 (2015).

## Supplementary Material

Supplementary Information Supplementary Figures 1-12, Supplementary Tables 1-4.

## Figures and Tables

**Figure 1:**
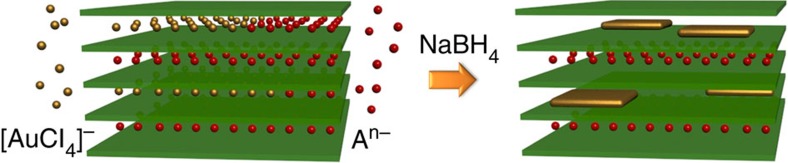
Schematic illustration of the synthetic procedure of 2D Au NSs. Green slices, golden squares and red/golden spheres represent LDH layers, 2D Au NSs and A^n−^/AuCl_4_^−^ anions, respectively.

**Table 1:** Catalytic data in catalytic oxidation of ethylbenzene and toluene.

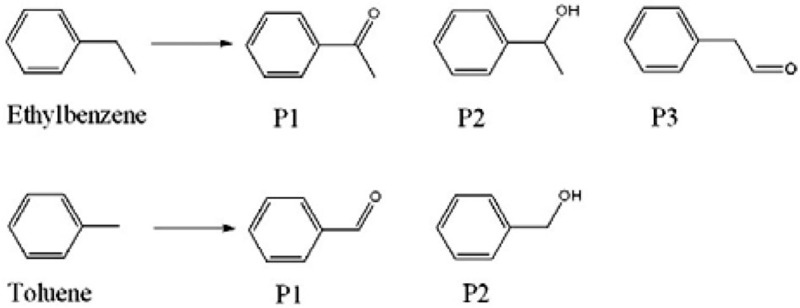								
**Entry**	**Subs.**	**Catalyst**	**Au (wt%)**	**Conv. (%)**	**Product selectivity (%)**			
					**P1**	**P2**	**P3**	Others*
1	PhEt	Au/LDH hybrid	0.13	39.2	91.0	4.0	1.8	3.2
2†		Au/LDH hybrid	0.13	32.5	91.9	5.8	—	2.3
3‡		Au/LDH hybrid	0.14	40.0	90.0	7.0	—	3.0
4		AuNP/FeO_x_	0.15	6.7	82.7	6.9	4.0	6.4
5		AuNP/C	0.20	Trace	—	—	—	—
6		AuNP/SiO_2_	0.15	0.5	—	—	—	—
7		AuNP/LDH	0.16	11.0	88.9	3.3	3.5	4.3
8	PhMe	Au/LDH hybrid	0.13	9.2	66.0	34.0	—	—
9		AuNP/LDH	0.16	1.3	90.2	9.8	—	—
10		AuNP/FeO_x_	0.15	Trace	—	—	—	—
11		AuNP/C	0.20	Trace	—	—	—	—
12		AuNP/SiO_2_	0.15	Trace	—	—	—	—

Conv., conversion; LDH, layered double hydroxide; Subs., substrate.

Reaction conditions: 47 mmol of substrate, 100 mg of catalyst, 16 h, 140 °C, oxygen pressure at 3 MPa. *t-*butyl hydroperoxide (TBHP, 3 mol% relative to the substrate) is added as an initiator^49^,^50^. The results of the reactions without using TBHP are given in Supplementary Table 2.

^*^2-phenylethanol, benzyl alcohol and others.

^†^Air was used as the oxidant, reaction time 24 h.

^‡^The catalyst was treated at 350 °C for 2 h before use.

## References

[b1] Seo J. W. . Two-dimensional nanosheet crystals. Angew. Chem. Int. Ed. 46, 8828–8831 (2007). 10.1002/anie.200703175 17939146

[b2] Kibsgaard J., Chen Z. B., Reinecke B. N. & Jaramillo T. F. Engineering the surface structure of MoS_2_ to preferentially expose active edge sites for electrocatalysis. Nat. Mater. 11, 963–969 (2012). 23042413 10.1038/nmat3439

[b3] Yuan W. J. . The edge- and basal-plane-specific electrochemistry of a single-layer graphene sheet. Sci. Rep. 3, 2248 (2013). 23896697 10.1038/srep02248 PMC3727060

[b4] Chen Z. B. . Core-shell MoO_3_-MoS_2_ nanowires for hydrogen evolution: a functional design for electrocatalytic materials. Nano Lett. 11, 4168–4175 (2011). 21894935 10.1021/nl2020476

[b5] Merki D., Fierro S., Vrubel H. & Hu X. L. Amorphous molybdenum sulfide films as catalysts for electrochemical hydrogen production in water. Chem. Sci. 2, 1262–1267 (2011).

[b6] Li Y. G. . MoS2 nanoparticles grown on graphene: an advanced catalyst for the hydrogen evolution reaction. J. Am. Chem. Soc. 133, 7296–7299 (2011). 21510646 10.1021/ja201269b

[b7] Jaramillo T. F. . Identification of active edge sites for electrochemical H-2 evolution from MoS_2_ nanocatalysts. Science 317, 100–102 (2007). 17615351 10.1126/science.1141483

[b8] Wang Q. H., Kalantar-Zadeh K., Kis A., Coleman J. N. & Strano M. S. Electronics and optoelectronics of two-dimensional transition metal dichalcogenides. Nat. Nanotechnol. 7, 699–712 (2012). 23132225 10.1038/nnano.2012.193

[b9] Lee Y. Y. . Top laminated graphene electrode in a semitransparent polymer solar cell by simultaneous thermal annealing/releasing method. ACS Nano. 5, 6564–6570 (2011). 21749099 10.1021/nn201940j

[b10] Lin T. Q., Huang F. Q., Liang J. & Wang Y. X. A facile preparation route for boron-doped graphene, and its CdTe solar cell application. Energy Environ. Sci. 4, 862–865 (2011).

[b11] Xiang Q. J., Yu J. G. & Jaroniec M. Synergetic effect of MoS2 and graphene as cocatalysts for enhanced photocatalytic H-2 production activity of TiO_2_ nanoparticles. J. Am. Chem. Soc. 134, 6575–6578 (2012). 22458309 10.1021/ja302846n

[b12] Li Q. . Highly efficient visible-light-driven photocatalytic hydrogen production of CdS-cluster-decorated graphene nanosheets. J. Am. Chem. Soc. 133, 10878–10884 (2011). 21639097 10.1021/ja2025454

[b13] Hamm J. M. & Hess O. Two two-dimensional materials are better than one. Science 340, 1298–1299 (2013). 23766321 10.1126/science.1239501

[b14] Shi Y. F. . Highly ordered mesoporous crystalline MoSe_2_ material with efficient visible-light-driven photocatalytic activity and enhanced lithium storage performance. Adv. Funct. Mater. 23, 1832–1838 (2013).

[b15] Iwase A., Ng Y. H., Ishiguro Y., Kudo A. & Amal R. Reduced graphene oxide as a solid-state electron mediator in Z-scheme photocatalytic water splitting under visible light. J. Am. Chem. Soc. 133, 11054–11057 (2011). 21711031 10.1021/ja203296z

[b16] Meyer J. C. . The structure of suspended graphene sheets. Nature 446, 60–63 (2007). 17330039 10.1038/nature05545

[b17] Novoselov K. S. . Electric field effect in atomically thin carbon films. Science 306, 666–669 (2004). 15499015 10.1126/science.1102896

[b18] Radisavljevic B., Radenovic A., Brivio J., Giacometti V. & Kis A. Single-layer MoS_2_ transistors. Nat. Nanotechnol. 6, 147–150 (2011). 21278752 10.1038/nnano.2010.279

[b19] Eda G. . Photoluminescence from chemically exfoliated MoS_2_. Nano Lett. 11, 5111–5116 (2011). 22035145 10.1021/nl201874w

[b20] Oyer A. J. . Stabilization of graphene sheets by a structured benzene/hexafluorobenzene mixed solvent. J. Am. Chem. Soc. 134, 5018–5021 (2012). 22413861 10.1021/ja211225p

[b21] Wang H. L., Robinson J. T., Li X. L. & Dai H. J. Solvothermal reduction of chemically exfoliated graphene sheets. J. Am. Chem. Soc. 131, 9910–9911 (2009). 19580268 10.1021/ja904251p

[b22] Coleman J. N. . Two-dimensional nanosheets produced by liquid exfoliation of layered materials. Science 331, 568–571 (2011). 21292974 10.1126/science.1194975

[b23] Chen S. H. & Carroll D. L. Synthesis and characterization of truncated triangular silver nanoplates. Nano Lett. 2, 1003–1007 (2002).

[b24] Bradley J. S., Tesche B., Busser W., Masse M. & Reetz R. T. Surface spectroscopic study of the stabilization mechanism for shape-selectively synthesized nanostructured transition metal colloids. J. Am. Chem. Soc. 122, 4631–4636 (2000).

[b25] Jiu J. T., Suganuma K. & Nogi M. Effect of additives on the morphology of single-crystal Au nanosheet synthesized using the polyol process. J. Mater. Sci. 46, 4964–4970 (2011).

[b26] Wu Y. W., Hang T., Wang N., Yu Z. Y. & Li M. Highly durable non-sticky silver film with a microball-nanosheet hierarchical structure prepared by chemical deposition. Chem. Commun. 49, 10391–10393 (2013). 10.1039/c3cc45592k 24072017

[b27] Duan H. H. . Ultrathin rhodium nanosheets. Nat. Commun. 5, 3093 (2014). 24435210 10.1038/ncomms4093

[b28] Banu K. & Shimura T. Synthesis of large-scale transparent gold nanosheets sandwiched between stabilizers at a solid-liquid interface. N. J. Chem. 36, 2112–2120 (2012).

[b29] Huang X. . Synthesis of hexagonal close-packed gold nanostructures. Nat. Commun. 2, 292 (2011). 21522136 10.1038/ncomms1291

[b30] Nootchanat S., Thammacharoen C., Lohwongwatana B. & Ekgasit S. Formation of large H_2_O_2_-reduced gold nanosheets via starch-induced two-dimensional oriented attachment. RSC Adv. 3, 3707–3716 (2013).

[b31] Huang X. . Synthesis of gold square-like plates from ultrathin gold square sheets: the evolution of structure phase and shape. Angew. Chem. Int. Ed. 50, 12245–12248 (2011). 10.1002/anie.201105850 22052613

[b32] Parker S. C. & Campbell C. T. Reactivity and sintering kinetics of Au/TiO_2_(110) model catalysts: particle size effects. Top. Catal. 44, 3–13 (2007).

[b33] Chen M. S. & Goodman D. W. The structure of catalytically active gold on titania. Science 306, 252–255 (2004). 15331772 10.1126/science.1102420

[b34] Valden M., Lai X. & Goodman D. W. Onset of catalytic activity of gold clusters on titania with the appearance of nonmetallic properties. Science 281, 1647–1650 (1998). 9733505 10.1126/science.281.5383.1647

[b35] Liu L. . The 2-D growth of gold on single-layer graphene/Ru(0001): enhancement of CO adsorption. Surf. Sci. 605, L47–L50 (2011).

[b36] Zhou Z. H., Gao F. & Goodman D. W. Deposition of metal clusters on single-layer graphene/Ru(0001): factors that govern cluster growth. Surf. Sci. 604, L31–L38 (2010).

[b37] Trifiro F. & Vaccari A. in Comprehensive Supramolecular Chemistry Vol. **7** 251Pergamon Elsevier Science (1996).

[b38] Sels B. . Layered double hydroxides exchanged with tungstate as biomimetic catalysts for mild oxidative bromination. Nature 400, 855–857 (1999).

[b39] Zhao M. Q. . Embedded high density metal nanoparticles with extraordinary thermal stability derived from guest-host mediated layered double hydroxides. J. Am. Chem. Soc. 132, 14739–14741 (2010). 20923190 10.1021/ja106421g

[b40] Ma R. Z., Liang J. B., Takada K. & Sasaki T. Topochemical synthesis of Co-Fe layered double hydroxides at varied Fe/Co ratios: unique intercalation of triiodide and its profound effect. J. Am. Chem. Soc. 133, 613–620 (2011). 21158476 10.1021/ja1087216

[b41] Kwon T., Tsigdinos G. A. & Pinnavaia T. J. Pillaring of layered double hydroxides (LDHs) by polyoxometalate anions. J. Am. Chem. Soc. 110, 3653–3654 (1988).

[b42] Thyveetil M. A., Coveney P. V., Greenwell H. C. & Suter J. L. Computer simulation study of the structural stability and materials properties of DNA-intercalated layered double hydroxides. J. Am. Chem. Soc. 130, 4742–4756 (2008). 18345669 10.1021/ja077679s

[b43] Song F. Q. . Free-standing graphene by scanning transmission electron microscopy. Ultramicroscopy 110, 1460–1464 (2010). 20850925 10.1016/j.ultramic.2010.09.001

[b44] Goris B. . Atomic-scale determination of surface facets in gold nanorods. Nat. Mater. 11, 930–935 (2012). 23085569 10.1038/nmat3462

[b45] Yu K., Wu Z. C., Zhao Q. R., Li B. X. & Xie Y. High-temperature-stable Au@SnO_2_ sore/shell supported catalyst for CO oxidation. J. Phys. Chem. C 112, 2244–2247 (2008).

[b46] Kesavan L. . Solvent-free oxidation of primary carbon-hydrogen bonds in toluene using Au-Pd alloy nanoparticles. Science 331, 195–199 (2011). 21233383 10.1126/science.1198458

[b47] Haruta M. Catalysis—gold rush. Nature 437, 1098–1099 (2005). 16237427 10.1038/4371098a

[b48] Quintanilla A. . Weakly bound capping agents on gold nanoparticles in catalysis: surface poison? J. Catal. 271, 104–114 (2010).

[b49] Hughes M. D. . Tunable gold catalysts for selective hydrocarbon oxidation under mild conditions. Nature 437, 1132–1135 (2005). 16237439 10.1038/nature04190

[b50] Zhao R. . A highly efficient oxidation of cyclohexane over Au/ZSM-5 molecular sieve catalyst with oxygen as oxidant. Chem. Commun. 904–905 (2004). 10.1039/b315098d 15045122

