# Supplementary material for: Two-dimensional gold nanostructures with high activity for selective oxidation of carbon–hydrogen bonds
Source: Nat Commun. 2015 Apr 22;6:6957. doi: 10.1038/ncomms7957 (PMC4421807; doi:10.1038/ncomms7957)
Supplement: Supplementary Information — Supplementary Figures 1-12, Supplementary Tables 1-4. [file ncomms7957-s1.pdf]

Supplementary Information

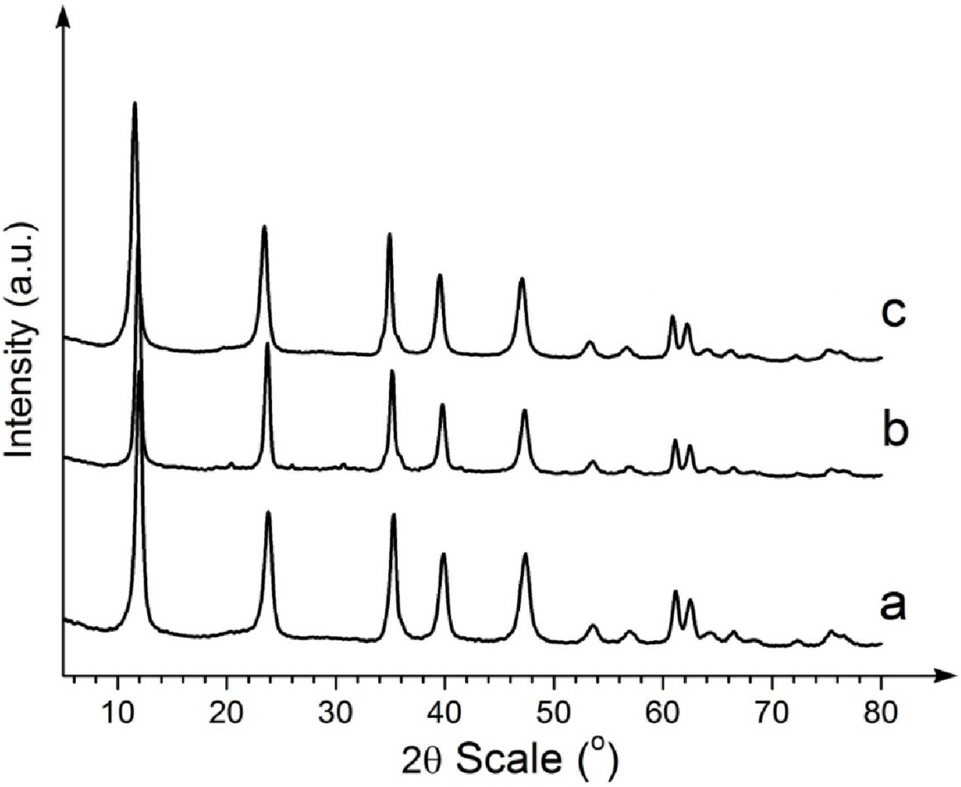

Supplementary Figure 1. X-ray diffraction patterns of (a) pure LDH, (b) $AuCl_4^-$ ion-exchanged LDH and (c) the Au/LDH hybrid catalyst. The refined cell parameters for pure, ion-exchanged, and 2D Au intercalated LDH samples are: a = 3.038(4) Å, c = 22.610(23) Å; a = 3.035(2) Å, c = 22.614(13) Å; and a = 3.038(2) Å, c = 22.640(15) Å respectively. The sample displacement effect has been taken into account for the XRD refinement. This indicates that such a small loading amount (0.13 wt%) of Au did not lead to considerable alteration in the unit cell parameters of the LDH support.

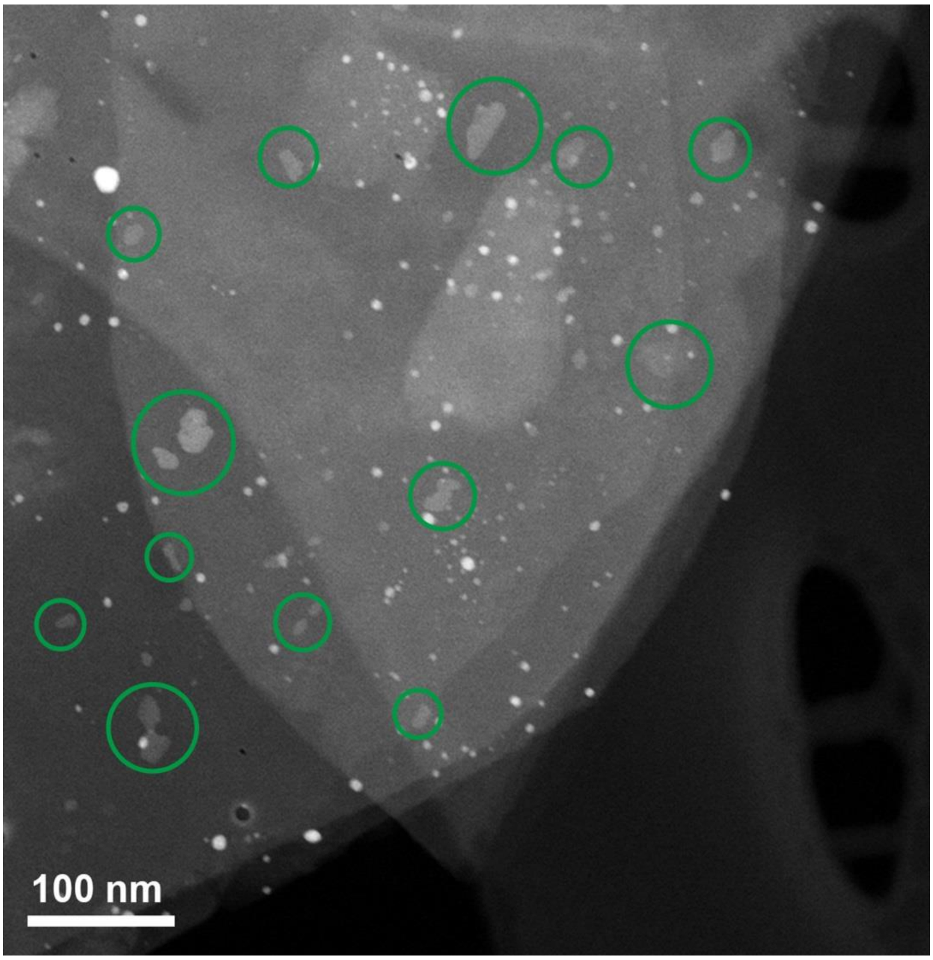

Supplementary Figure 2. HAADF-STEM image of the as-synthesized Au/LDH hybrid. 2D Au NSs with brighter contrast as confirmed by EDX are marked by green circles.

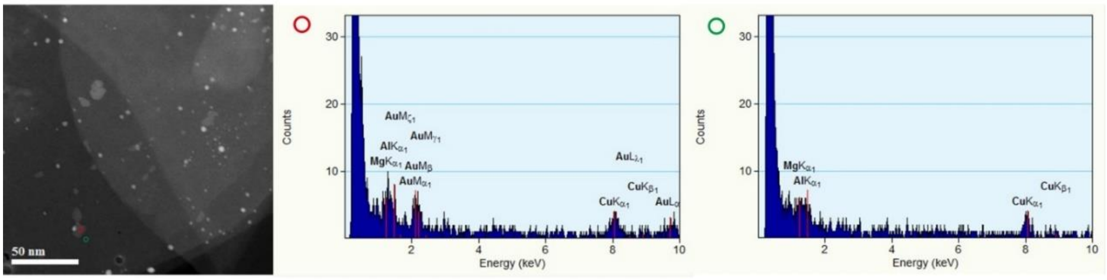

Supplementary Figure 3. HAADF-STEM image of the as-synthesized Au/LDH hybrid, and EDX spectra acquired from two selected areas with (red circle) and without (green circle) 2D Au NSs.

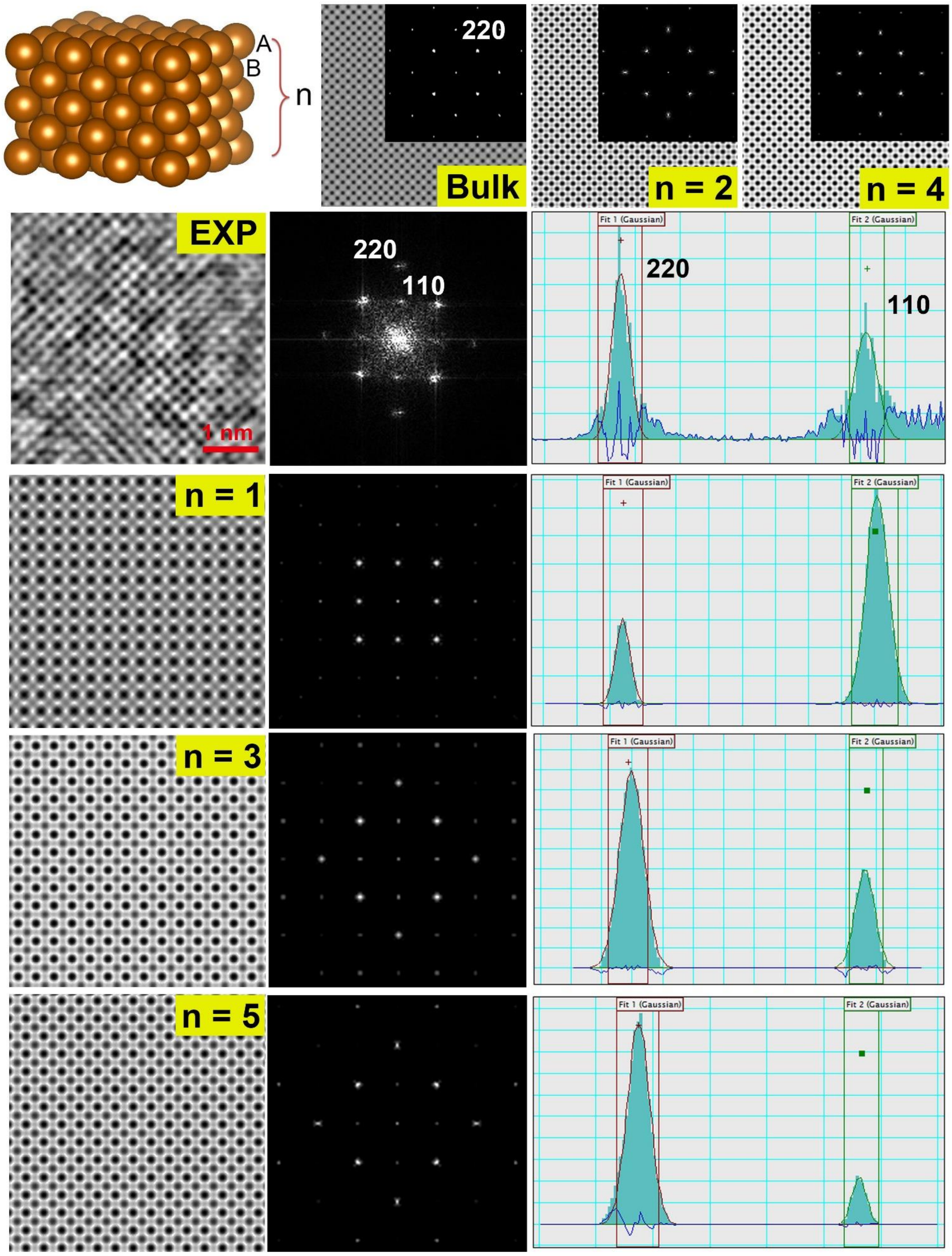
A
B
n
220
Bulk
n = 2
n = 4
EXP
1 nm
220
110
Fit 1 (Gaussian)
Fit 2 (Gaussian)
220
110
n = 1
Fit 1 (Gaussian)
Fit 2 (Gaussian)
n = 3
Fit 1 (Gaussian)
Fit 2 (Gaussian)
n = 5
Fit 1 (Gaussian)
Fit 2 (Gaussian)

Supplementary Figure 4. Contrast-Transfer-Function (CTF) correction and dynamical simulation of HRTEM image and FFT diffractogram of [001] oriented (*i.e.*, “AB” stacked) 2D Au NSs with different thicknesses (denoted by the layer number “n”). The average thickness of 2D Au NSs with even a few nanometer lateral dimensions can be determined from the quantitative analysis of the diffractogram derived via a fast Fourier transform (FFT) of HRTEM images once the oscillating CTF is corrected. The spherical-aberration and astigmatism-induced image distortions are eliminated via the CTF/astigmatism correction by reconstructing the through-focus series of 20 HRTEM images taken on a [001] oriented 2D Au NS with a focus step of -5 nm and a starting focus of -35.1 nm (determined during reconstruction, refers to *Ultramicroscopy*, 115, **2012**, 50). The corrected image (denoted as “EXP”) best represents the structural projection with a resolution up to the information limit of the microscope. The FFT diffractograms of the experimentally reconstructed and a series of simulated HRTEM images of 2D Au NSs with different thicknesses are compared (simulation conditions: 300 kV, Cs = 0 mm, Δf = 0 nm and a focal spread of 4 nm), where the amplitudes of the (220) and (110) reflections are extracted and profiled (10 pixel width). The relative intensity of (220) and (110) reflections critically depends on the average thickness of the [001] oriented 2D Au NSs. For thicker (e.g., denoted as “bulk”) NSs and those with an even layer number, the (110) reflections are absent due to the lattice symmetry. The (110) reflections only appear in those few-layer NSs with an odd layer number and the intensity ratio of (110)/(220) reflections will dramatically decrease when the layer number increase. Based on the investigations of both experimental and simulated FFT diffractograms, it is concluded that the observed [001] oriented 2D Au NS may have three layers.

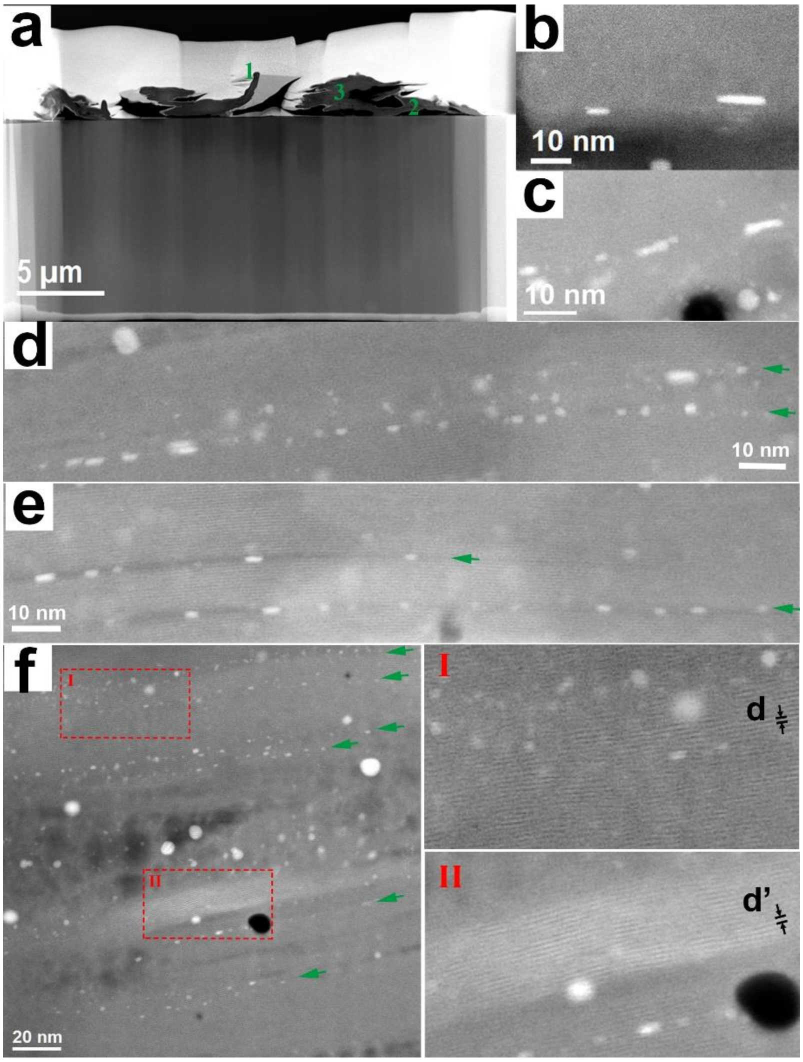

Supplementary Figure 5. (a) Low-magnification HAADF-STEM image of the FIB cross-sectioned slice of the Au/LDH hybrid. (b~f) High-resolution images, showing elongated Au particles and intercalated Au nanosheets. The arrows indicate linear arrays of Au particles, which are likely formed as a consequence of the breaking of 2D Au nanosheets. (b) is from region 1, (c~e) are from region 2, and (f) is from region 3 in (a).

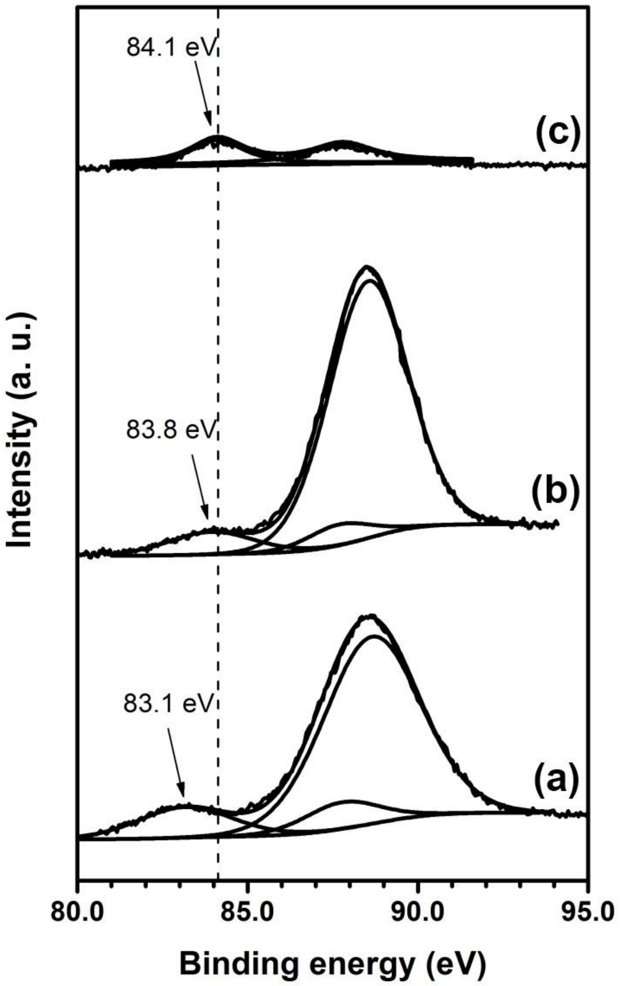

Supplementary Figure 6. Au 4f XPS spectra of different catalysts: (a) the Au/LDH hybrid, (b) AuNP/LDH, and (c) AuNP/SiO_2. The spectra include both Au 4f and Al 2p peaks that are deconvoluted using a mixed Gaussian-Lorentzian profile.

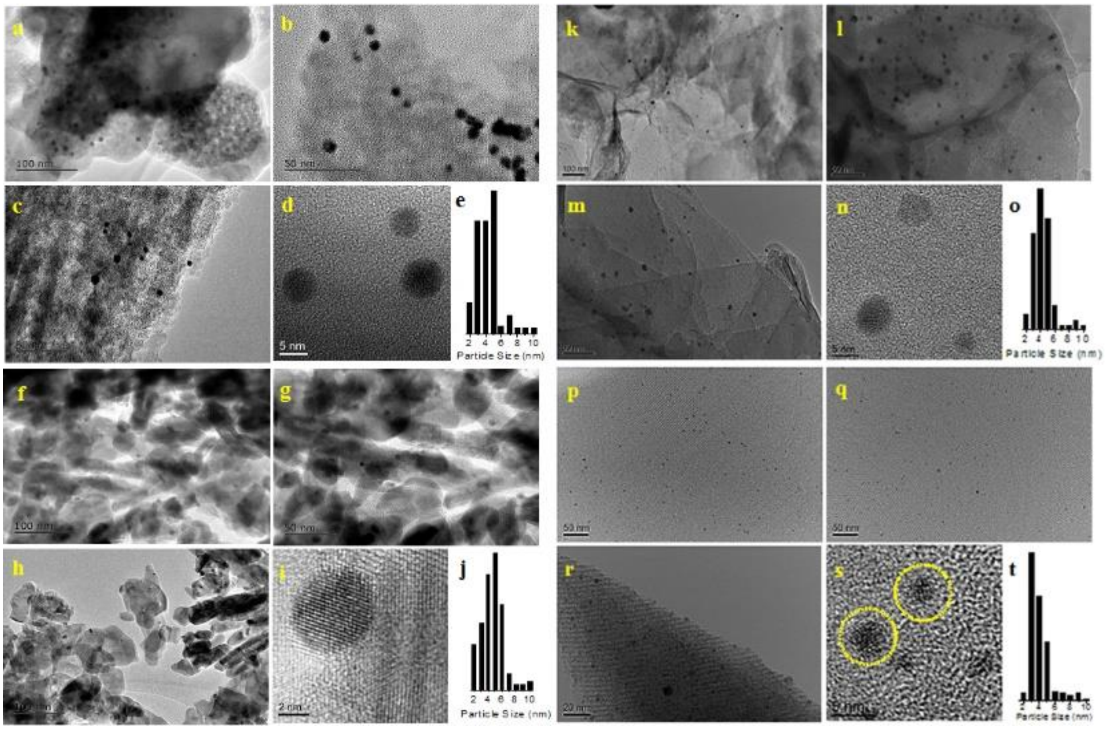

Supplementary Figure 7. Representative TEM images at different magnifications and the size distributions of Au nanoparticles in different catalysts: (a~e) AuNP/C, (f~j) AuNP/FeO_x, (k~o) AuNP/LDH, (p~t) AuNP/SiO_2. The particle size distributions for each catalyst were derived from more than 180 Au nanoparticles over multiple TEM images.

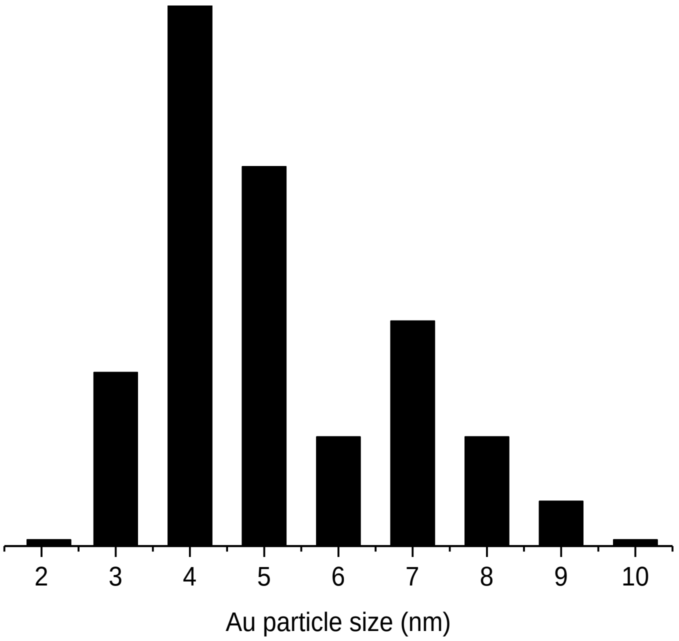

Supplementary Figure 8. Particle size distribution of Au nanoparticles in the Au/LDH hybrid based on TEM images.

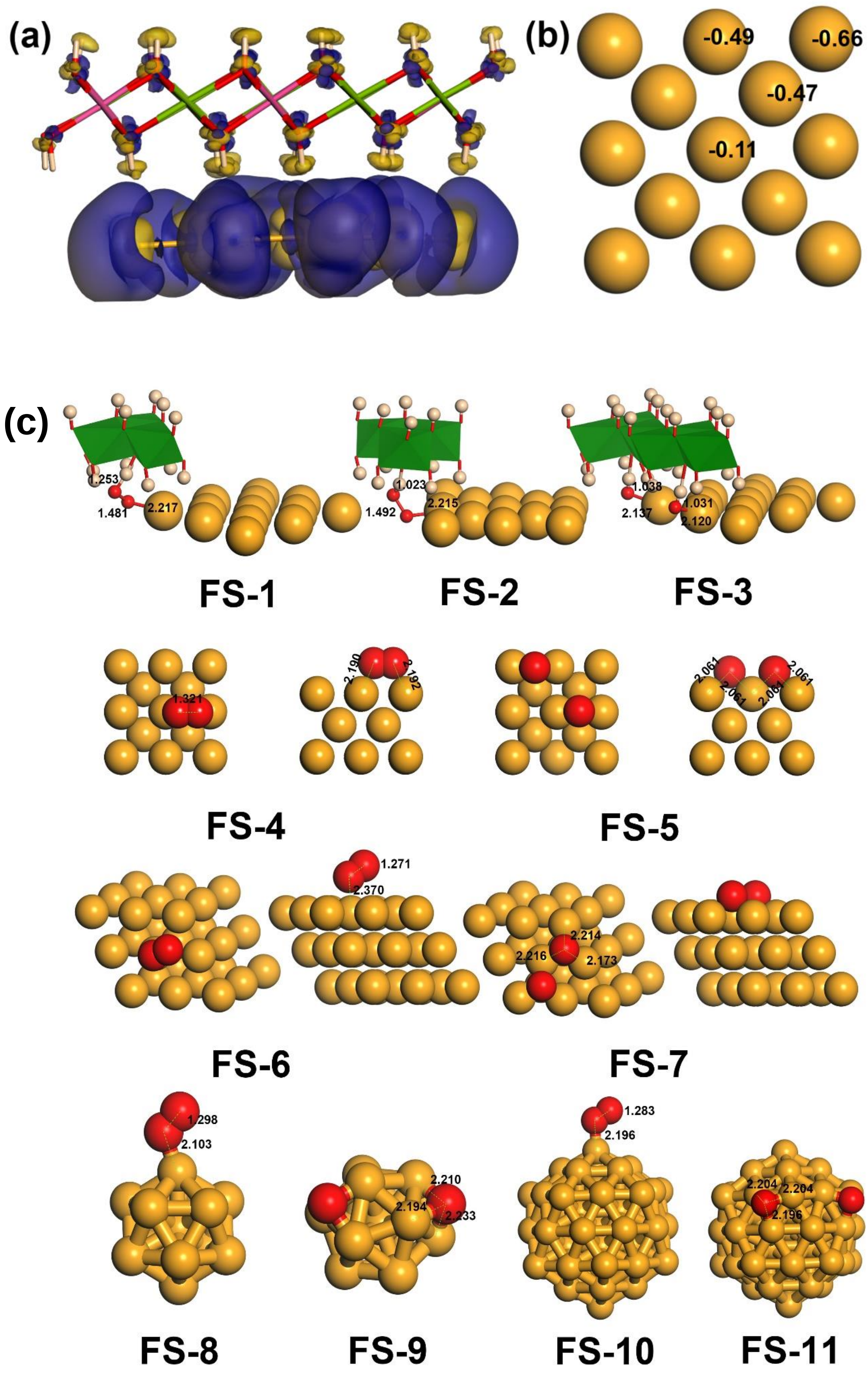
(a)
(b)
-0.49
-0.66
-0.47
-0.11
(c)
1.253
1.481
2.217
1.023
1.492
2.215
1.038
2.137
1.031
2.120
FS-1
FS-2
FS-3
1.321
2.190
2.192
2.061
FS-4
FS-5
1.271
2.370
2.214
2.216
2.173
FS-6
FS-7
1.298
2.103
2.210
2.194
2.233
1.283
2.196
2.204
2.204
2.196
FS-8
FS-9
FS-10
FS-11

Supplementary Figure 9. (a) The charge density difference isosurface of 2D Au_{13}/LDH interface, revealing electron surplus (blue) in 2D Au_{13} and depletion (yellow) in LDH framework. (b) Mulliken charge population of symmetry-inequivalent sites. (c) Optimized geometries of oxygen adsorbed on Au model structures. For clarity, the LDH framework is not entirely shown.

The analysis of both the electron density difference and Mulliken charge revealed the electron transfer from the LDH framework to the 2D Au_{13} cluster and the negative charging of the cluster (Figures S9a and S9b), in good agreement with the XPS results. Combined with the calculated molecular and dissociative adsorption energies for various Au surfaces and clusters (Figure S9c and Table S3), the exposed edge sites of the negatively charged 2D Au_{13} cluster have the largest adsorption energy for both molecular and dissociative adsorption. It is consistent with the literature that negatively charged Au adsorbs oxygen more strongly than do neutral ones. Interestingly, we found that the hydroxyl groups of the LDH cationic layer in the vicinity of 2D Au_{13} cluster can play a synergetic role in activating the oxygen by bonding to the adsorbed oxygen species, further enhancing the adsorption of oxygen onto the 2D Au_{13}/LDH hybrid structure. The calculated O-O distance of activated molecular oxygen species on the 2D Au_{13}/LDH hybrid is much longer than those on neutral Au surfaces and clusters, and is characteristic of typical peroxide species. Such species may be a key intermediate for the activation C-H bonds in various substrates and are only formed via the cooperative activation of oxygen by both the hydroxyl group in LDH and 2D Au at their interfaces. Similarly, hydroxyl species bonded to 2D Au are formed if the oxygen is activated via a dissociative pathway. On the basis of these results, we infer that the unique oxidation activity of Au/LDH hybrid is associated with its strong capability to activate oxygen both molecularly and dissociatively.

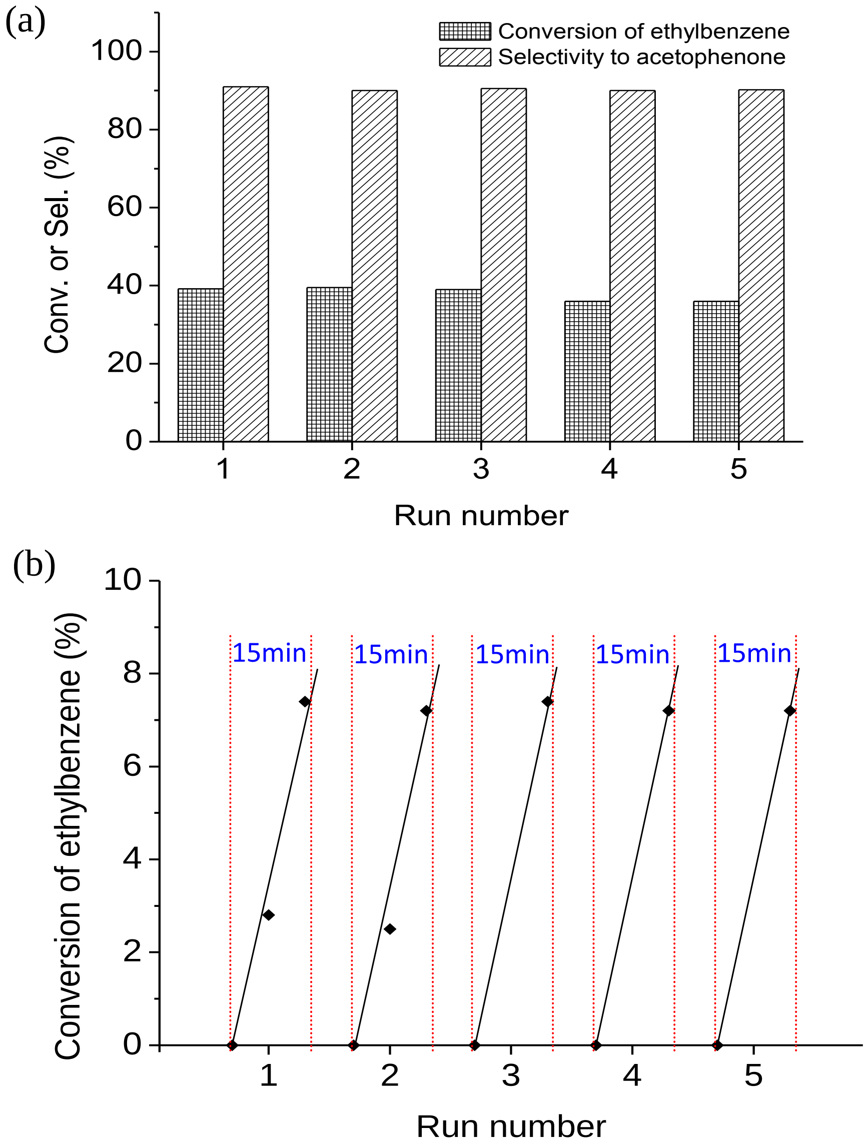

Supplementary Figure 10. Recycling results of the aerobic oxidation of ethylbenzene over the Au/LDH hybrid catalyst. The reaction conditions are the same to those described in Table 1. (a) Conversion of ethylbenzene and selectivity to acetophenone in each run; (b) Conversion of ethylbenzene at the beginning (15 min) of each run. The black straight lines are made by fitting the conversion points. Clearly, these straight lines have similar slopes, suggesting the similar conversion rate in each run.

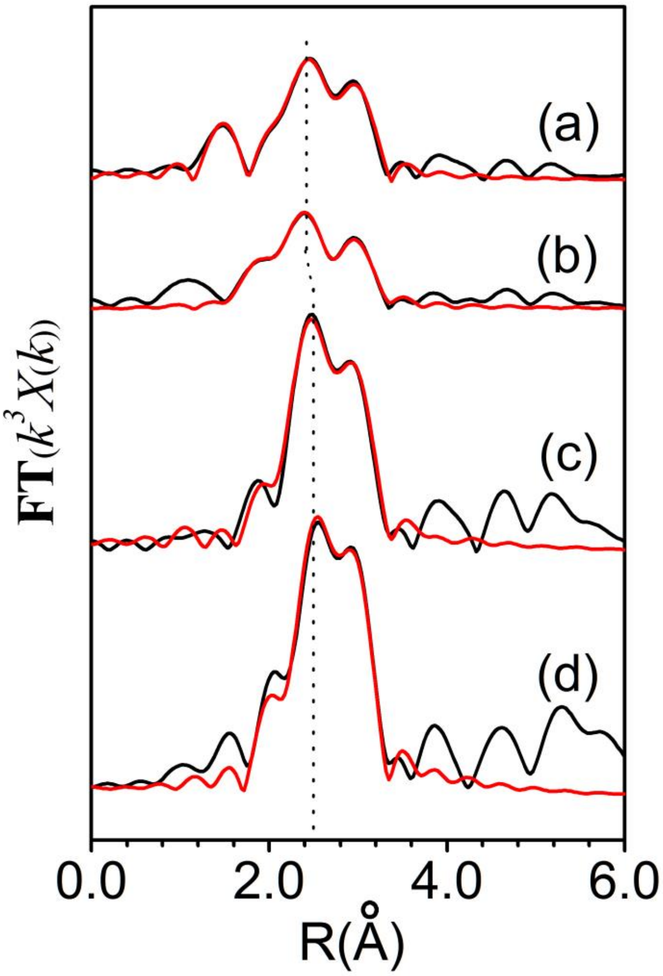

Sample	Reference	Shell	CN †	R ‡ (Å)	DW⊥ (Å^2)	R factor (%)
**2D Au**	Au	Au-Au	7.7 ± 1.1	2.85 ±0.02	0.001	1.6
		Au-O	0.5 ± 0.1	1.94 ±0.02	0.011	
**2D Au-350**	Au	Au-Au	7.5 ± 1.3	2.83 ±0.02	0.002	0.2
		Au-O	0.2 ± 0.1	2.18 ±0.02	0.014	
**2D Au-550**	Au	Au-Au	10.2±1.6	2.85 ±0.02	0.008	0.6
**2D-Au-Diss**	Au	Au-Au	10.3±0.8	2.86±0.01	0.008	0.5

† CN: coordination number. ‡ R: bond distance. ⊥ DW: Debye-Waller factor.

Supplementary Figure 11. EXAFS spectra by Fourier transforms of filtered $k^3 \cdot \chi(k)$ into the R space of the Au-L_{III} edge in (a) as-synthesized Au/LDH hybrid nanosheets, (b) 350 ºC calcined, (c) 550 ºC calcined and (d) Au species collected from the Au/LDH hybrid by dissolving the LDH support with HCl. It is shown that upon the removal of LDH, 2D NSs would transform into Au NPs. The red dashed lines correspond to the curve-fitting results. The fitted structural parameters from EXAFS spectra, e.g., average coordination number and bond distance, are compiled in the Table. Note that the result of the as-synthesized Au/LDH hybrid shown here is from an independent EXAFS experiment from that discussed in Fig. 4 and Table S1. The results from two independent experiments are well consistent in both the refined Au-Au coordination number and bond distance.

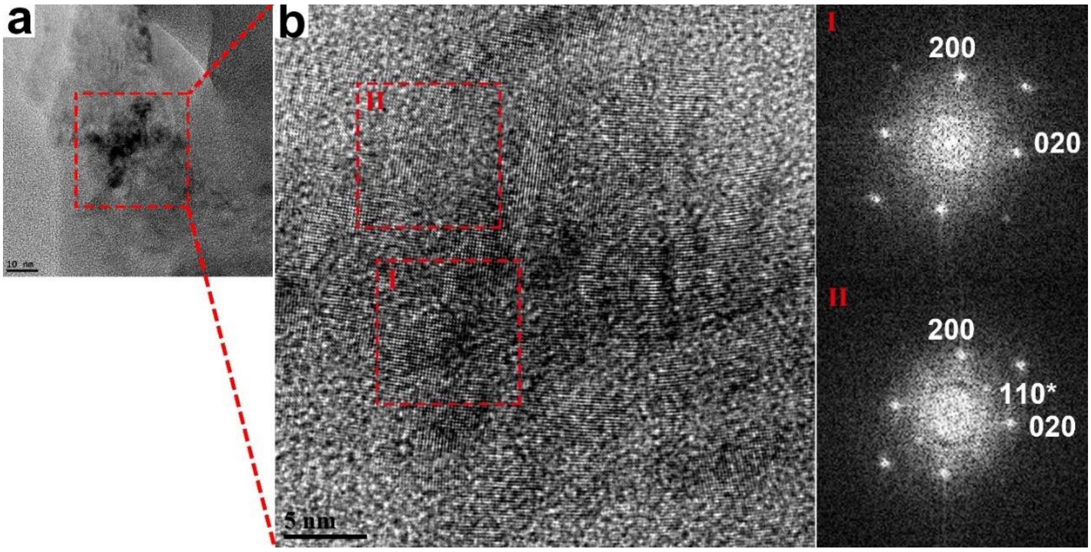

Supplementary Figure 12. (a) low-magnification TEM and (b) HRTEM images of a Au/LDH hybrid sample after a thermal treatment (350 °C, 2 h) and catalytic use. The highlighted area in (a) corresponds to a 2D Au nanosheet. On the right are the FFT diffractograms derived from the two square regions in (b) as labelled. The forbidden (110) reflections appear in the FFT of region II, indicating the ultra-thin nature of the sheet. The absence of (110) reflections in the FFT of region I implies a difference in thickness (number of atomic layers) between the two regions (refer to the discussion of Supplementary Figure 4).

Supplementary Table 1. EXAFS parameters of Au/LDH hybrid and Au/SiO_2 samples.

Sample	Reference	Shell	CN†	R ‡ (Å)	DW$^{\perp}$ (Å)	R factor (%)
Au foil	Au	Au-Au	11.2 ± 0.9	2.85 ±0.01	0.090	0.6
Au/LDH hybrid	Au	Au-Au	6.9 ± 0.6	2.80 ±0.01	0.117	0.6
		Au-O	0.7 ± 0.3	2.11 ±0.04	0.109	
Au/SiO_2	Au	Au-Au	10.1 ± 0.8	2.86 ±0.01	0.093	0.7

† Coordination number. ‡ Bond distance. $^{\perp}$Debye-Waller factor.

Supplementary Table 2. Catalytic results of various catalysts in oxidation of ethylbenzene under different conditions.

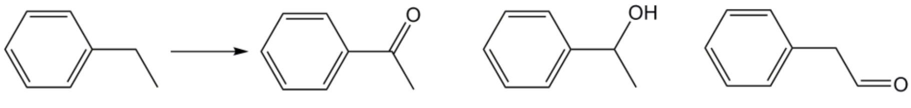

Entry	Catalyst	Reaction conditions	Conv. (%)	Product selectivity (%)			
				P1	P2	P3	Others
1	LDH	TBHP, O_2 †	Trace	--	--	--	--
2	Au/LDH hybrid	TBHP, O_2 †	39.2	91.0	4.0	1.8	3.2
3	AuNP/LDH	TBHP, O_2 †	11.0	88.9	3.3	3.5	4.3
4	AuNP/FeO_x	TBHP, O_2 †	6.7	82.7	6.9	4.0	6.4
5	Au/LDH hybrid	O_2 ‡	5.3	88.9	8.0	2.1	--
6	AuNP/LDH	O_2 ‡	Trace	--	--	--	--
7	AuNP/FeO_x	O_2 ‡	Trace	--	--	--	--

† Entry 2 to 4 are adopted from Table 1. Reaction conditions: 47 mmol of substrate, 100 mg of catalyst, 16 h, 140 °C, oxygen pressure at 3 MP, *t*-butyl hydroperoxide (TBHP, 3 mol% based on substrate) is added as initiator.

‡ Reaction conditions: 47 mmol of substrate, 100 mg of catalyst, 16 h, 140 °C, oxygen pressure at 3 MP, no TBHP was added.

Supplementary Table 3. Calculated Energies for Molecular and Dissociative Adsorption (MA and DA) of O_2 to Various Au Surfaces and Clusters†

Substrate	Geometry	Mode	Site	E_{ads} (eV)	$d_{O\text{-}O}$ (Å)
$Au_{13}(D_{4h})$/LDH	FS-1	MA1	Corner	2.21	1.480
	FS-2	MA2	Edge	2.45	1.492
	FS-3	DA	Edge	3.35	
Au(100)	FS-4	MA	Bridge	0.79	1.321
	FS-5	DA	Bridge	0.75	
Au(111)	FS-6	MA	Atop	0.17	1.271
	FS-7	DA	Hollow	-0.78	
$Au_{13}(I_h)$	FS-8	MA	Atop	0.79	1.298
	FS-9	DA	Hollow	0.54	
$Au_{55}(I_h)$	FS-10	MA	Atop	0.98	1.283
	FS-11	DA	Hollow	1.10	

† Computational Methods: The *ab initio* DFT calculation was carried out by CASTEP codes, which was generally based on plane-wave pseudopotential method. The wave functions of the valance electrons were expanded using a plane wave basis set within a specified cutoff energy of 340.0 eV. The core electrons were treated by ultra-soft plane-wave pseudopotentials (USP) approach represented in the reciprocal space which also include the nonlinear core correction (NLCC) for the more precise treatment of possible semicore electrons. Perdew-Burke-Ernzerhof (PBE) was used as the exchange-correlation (XC) functional with general-gradient approximations (GGA). The density mixing method and the BFGS geometry optimization method are used to optimize the electronic structure and the atomic configurations, respectively. The ground state geometries were reached when maximum energy change, force and atomic displacement approached the 1.0×10^{-5} eV/atom, 0.03 eV/Å and 0.001 Å tolerances respectively. A model structure made of a 2D Au_{13} cluster with a D_{4h} point symmetry embedded in a 4×4×1 supercell of $[Mg_3Al(OH)_8]^+$ LDH cationic layers was employed. The corresponding oxygen molecular and dissociative adsorption energies,

as defined by $E_{ads} = E_{substrate} + E_{O2} - E_{substrate+Oads}$ were calculated, and compared with those of neutral (100)/(111) Au surfaces and magic-number clusters, i.e., Au_{13} and Au_{55} icosahedra with I_h point symmetry.

Supplementary Table 4. Solvent-free oxidation of various substrates over the Au/LDH hybrid catalyst.

Reactant	Product	Conv. (%)	Sel.[†] (%)
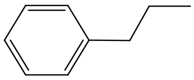	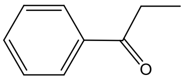	52.2	93.0
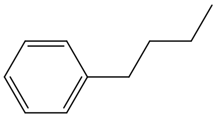	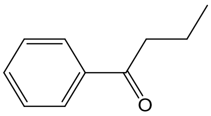	47.1	90.6
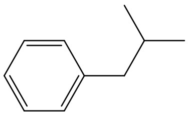	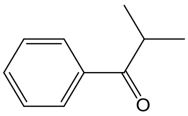	49.9	89.0
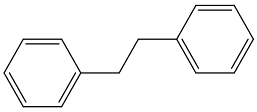	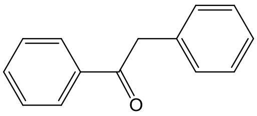	76.4[‡]	20.0
	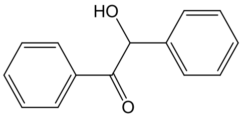		21.9
	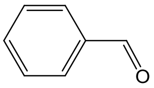		50.2
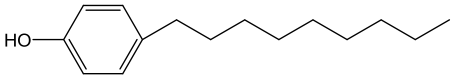	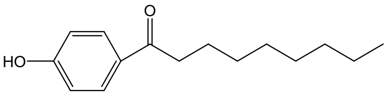	30.5[‡]	83.0
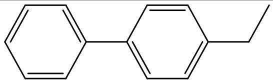	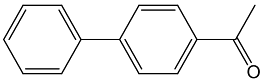	37.1	88.0
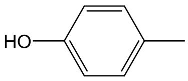	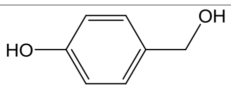	7.4	61.0
	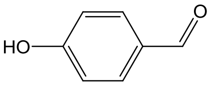		39.0

Reaction conditions: 47 mmol of substrate, 100 mg of catalyst, 16 h, 140 °C, oxygen pressure at 3 MP, *t*-butyl hydroperoxide (TBHP, 3 mol% based on substrate) is added as initiator.

[†] The by-products are alcohols, aldehydes, and some others.

[‡] Reaction temperature at 160 °C.
